# Role of Endothelial Cells in the Pathogenesis of Influenza in Humans

**DOI:** 10.1093/infdis/jiz349

**Published:** 2019-07-08

**Authors:** Kirsty R Short, Thijs Kuiken, Debby Van Riel

**Affiliations:** 1 School of Chemistry and Molecular Biosciences, University of Queensland, Brisbane, Australia; 2 Australian Infectious Diseases Research Centre, University of Queensland, Brisbane, Australia; 3 Department of Viroscience, Erasmus Medical Center, Rotterdam, The Netherlands

To the Editor—In the recent report by Chan and colleagues [1], human tissues and primary cells from the respiratory tract were used to determine the cell tropism and replication kinetics of low and highly pathogenic avian H7N9 influenza viruses. One of their key findings is that in contrast to low pathogenic H7N9, the highly pathogenic H7N9 virus replicated efficiently in human pulmonary microvascular endothelial cells, a feature previously only observed for highly pathogenic H5N1 viruses. In the discussion, the authors suggest that the infection of endothelial cells by highly pathogenic H7N9 and H5N1 viruses may contribute to the pathogenicity of the virus and dissemination of virus beyond the respiratory tract.

However, in mammals, including humans, there is limited in vivo evidence of productive influenza virus infection of endothelial cells. This is based on the fact that endothelial cells are hardly ever positive for influenza virus antigen in both human autopsy cases as well as experimental animal models after infection with either seasonal, pandemic, or zoonotic highly pathogenic influenza viruses [2]. In the cases where endothelial cell infection is observed, this does not exceed more than 2% of the pulmonary endothelial cells [3]. These observations contrast with the extensive infection of endothelial cells observed in poultry and swans (*Cygnus* species) after infection with highly pathogenic avian influenza (HPAI) viruses. Remarkably, this endothelial cell tropism is not observed in many other avian species, such as wild ducks [2], suggesting that endothelial cell tropism is a species-specific feature that cannot be extrapolated to all avian species or mammals, including humans [2]. An exception to this pattern is the widespread infection of endothelial cells in H5N1 virus–infected cats, but only after gastrointestinal inoculation [2].

Endothelial cells still may play a role in the pathogenesis of severe influenza, even if they do not support efficient influenza virus infection in vivo. As noted by Chan and colleagues, endothelial cells become activated during influenza virus infection, based on increasing levels of von Willebrand factor [4], increased coagulation [5], and necrosis [6]. In mice and ferrets infected with influenza virus, endothelial cells produce a variety of pro-inflammatory cytokines [2, 7, 8] that can be blocked by a SIP1 receptor agonist [7]. Interestingly, Chan et al observed increased endothelial cell inflammation following HPAI H5N1, rather than HPAI H7N9 infection, suggesting that this effect may be virus subtype specific.

It is poorly understood what triggers endothelial cell responses to influenza virus in vivo. In the alveoli of the lower respiratory tract, endothelial cells are in close proximity to alveolar epithelial cells, which are permissive for influenza virus infection. In some locations, epithelial and endothelial cells are separated only by a single basement membrane. It is therefore likely that infection of alveolar epithelial cells results in exposure of endothelial cells to virus particles, either via basolateral release of viruses from alveolar epithelial cells (as described by Chan and colleagues) or damage of the alveolar wall due to infection, necrosis, and inflammation. Instead of emphasizing the role of endothelial cells in virus replication, we propose that in vivo exposure to influenza virus particles results in an abortive infection in endothelial cells (Figure 1). This abortive infection, instead of augmenting viral replication, would trigger innate responses in endothelial cells and subsequent release of associated cytokines in the circulation. In this way, abortive infection of endothelial cells could explain the similar levels of messenger RNA independent of virus production (Chen et al) and the virus-induced pro-inflammatory response of endothelial cells in H5N1 virus–infected mice [3].

**Figure 1.**
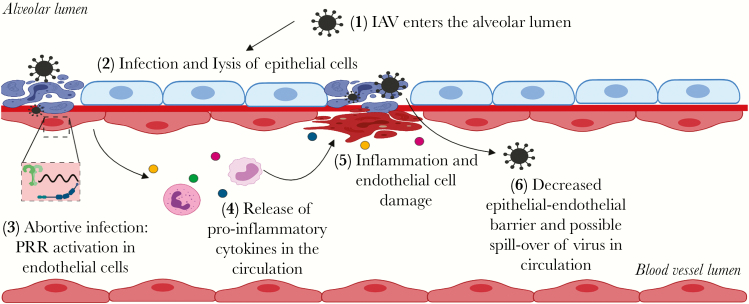
Schematic representation of the possible role of mammalian endothelial cells in influenza virus pathogenesis. IAV, influenza A virus; PRR, pattern recognition receptors. Figure created with BioRender.com.

Data are accumulating that severe influenza causes disease not only in the respiratory tract, but also in extra-respiratory tissues and even systemically. In experimentally infected ferrets, pro-inflammatory cytokines were induced in extra-respiratory tissues [8, 9], and in humans with influenza, higher levels of pro-inflammatory cytokines in the circulation are associated with higher morbidity and mortality [10]. In both situations, we speculate that abortive influenza virus infection in extra-respiratory tissues—in either endothelial cells, parenchymal cells, or both—may trigger pro-inflammatory responses and thus exacerbate the severity of disease from influenza virus infection.

## Notes

***Financial support.*** K. R. S. is supported by an Australian Research Council DECRA (grant number DE180100512), T. K. by DELTA-FLU European Union H2020-EU (grant number 727922), and D. v. R. by the Netherlands Organization for Scientific Research (grant number 91718308).

***Potential conflicts of interest.*** The authors: No reported conflicts of interest. All authors have submitted the ICMJE Form for Disclosure of Potential Conflicts of Interest. Conflicts that the editors consider relevant to the content of the manuscript have been disclosed.
